# DR region of Na^+^-K^+^-ATPase is a new target to protect heart against oxidative injury

**DOI:** 10.1038/s41598-018-31460-z

**Published:** 2018-08-30

**Authors:** Fei Hua, Zhiyuan Wu, Xiaofei Yan, Jin Zheng, Haijian Sun, Xu Cao, Jin-Song Bian

**Affiliations:** 1 Department of Pharmacology, Yong Loo Lin School of Medicine, National University of Singapore, Singapore, 117597 Singapore; 2 Department of Genetics and Molecular Biology, Medical College of Xi’an Jiaotong University, Xi’an, Shaanxi China; 3 Department of Kidney Transplant, the First Affiliated Hospital of Xi’an Jiaotong Unviersity, Xi’an, Shaanxi China; 4 National University of Singapore (Suzhou) Research Institute, Suzhou, China

## Abstract

Previous studies have shown that the activity and expression of Na^+^/K^+^-ATPase (NKA) are down-regulated in the failing hearts, and that an antibody against the DR-region of NKA (DR-Ab) can stimulate its activity. The present study was designed to investigate the beneficial effects of this antibody against cardiac injury and the underlying mechanisms. We found that DR-Ab improved cardiac function, alleviated cardiac hypertrophy and reduced oxidative stress in isoproterenol-treated mice. In AC16 human cardiomyocytes, DR-Ab increased cell viability and attenuated apoptosis under oxidative stress. Corresponding to the observation of reduced NKA activity, NKA abundance on plasma membrane was lowered during oxidative stress. Suppressed activity of protein phosphatase 2 A (PP2A) was responsible for the loss of membrane NKA due to the increased phosphorylation of key serine residues that trigger endocytosis. Incubation with DR-Ab restored PP2A activity and stabilized NKA expression on the plasma membrane. Inhibitors of PP2A abolished the protective effect of DR-Ab against oxidative stress. In summary, our data indicate that loss of membrane NKA may contribute to cardiac pathologies in heart failure. DR-Ab, by stabilizing membrane NKA, protects cardiomyocytes against oxidative injury and improves cardiac function in the failing hearts, suggesting a novel approach to treat heart failure.

## Introduction

In spite of the advancing knowledge in cardiac pathologies over the years, cardiovascular diseases remain a major cause of mortality and morbidity worldwide^1,2^. Oxidative stress is thought to be the main cause of the deterioration of cardiac function in patients^3^. Reactive oxygen species (ROS) are increased in various heart diseases including myocardial infarction, ischemia/reperfusion injury, hypertrophy and heart failure. High levels of ROS induce cell injuries, including necrosis and apoptosis. In hypertrophy, ROS activate pathological responses including reprogramming of gene expression, and an increase in protein synthesis^4^.

The Na^+^/K^+^-ATPase (NKA) is a ubiquitously expressed transmembrane protein that actively exchanges three Na^+^ out of and two K^+^ into cells^5^. This process is responsible for maintaining the electrochemical gradient, and hence the membrane potential, of the cell membrane. Recent evidence directly indicates that reduced NKA level may induce cardiomyocyte death and cardiac dysfunction^6^. Decreased NKA activity and expression have long been associated with heart failure in both animal models^7^ and human patients^8–10^. Another interesting observation is that heart failure patients are more sensitive to cardiac glycosides, a group of molecules commonly used to improve cardiac contractility by inhibiting the sodium pump, as a result of decreased NKA expression^6,11^. Together, reduced NKA activity and expression is clearly associated with the viability of cardiomyocytes and various cardiac conditions, making stabilization of NKA a plausible approach for cardioprotection.

Our group and others have previously reported that a rabbit polyclonal antibody (DR-Ab) which targets the extracellular region ^897^DVEDSYGQQWTYEQR^911^ (DR region) of M7/ M8 on NKA α subunit stimulates NKA activity and heart contractility by triggering Src/ERK1/2 pathway^12–14^. We also found that the same antibody confers cardioprotection against ischemic injury in both rat cardiomyocytes and isolated hearts, probably via the activation of PI3K/Akt/ERK pathway^12^. Given the significance of a stable NKA concentration in maintaining viable myocardium, and the central role of ROS in ischemia and reperfusion injury, we performed experiments to examine whether the protective effect of DR-Ab in failing hearts is mediated by maintaining functional NKA under oxidative stress and its molecular mechanisms.

The plasma NKA expression can be regulated by phosphorylation of critical species-specific serine residues on the NKA α-subunit^15–20^. Specifically, phosphorylation of NKA by PKCζ marks the trigger of endocytosis, while dephosphorylation by PP2A promotes recruitment and maintenance of NKA within the plasma membrane^16,21^ We hypothesize that DR-Ab may protect heart against oxidative stress by stabilization of NKA on the plasma membrane through activation of PP2A.

## Results

### Generation of DR-Ab and its therapeutic effects in an ISO-induced mouse cardiac hypertrophy model

**Figure 1:**
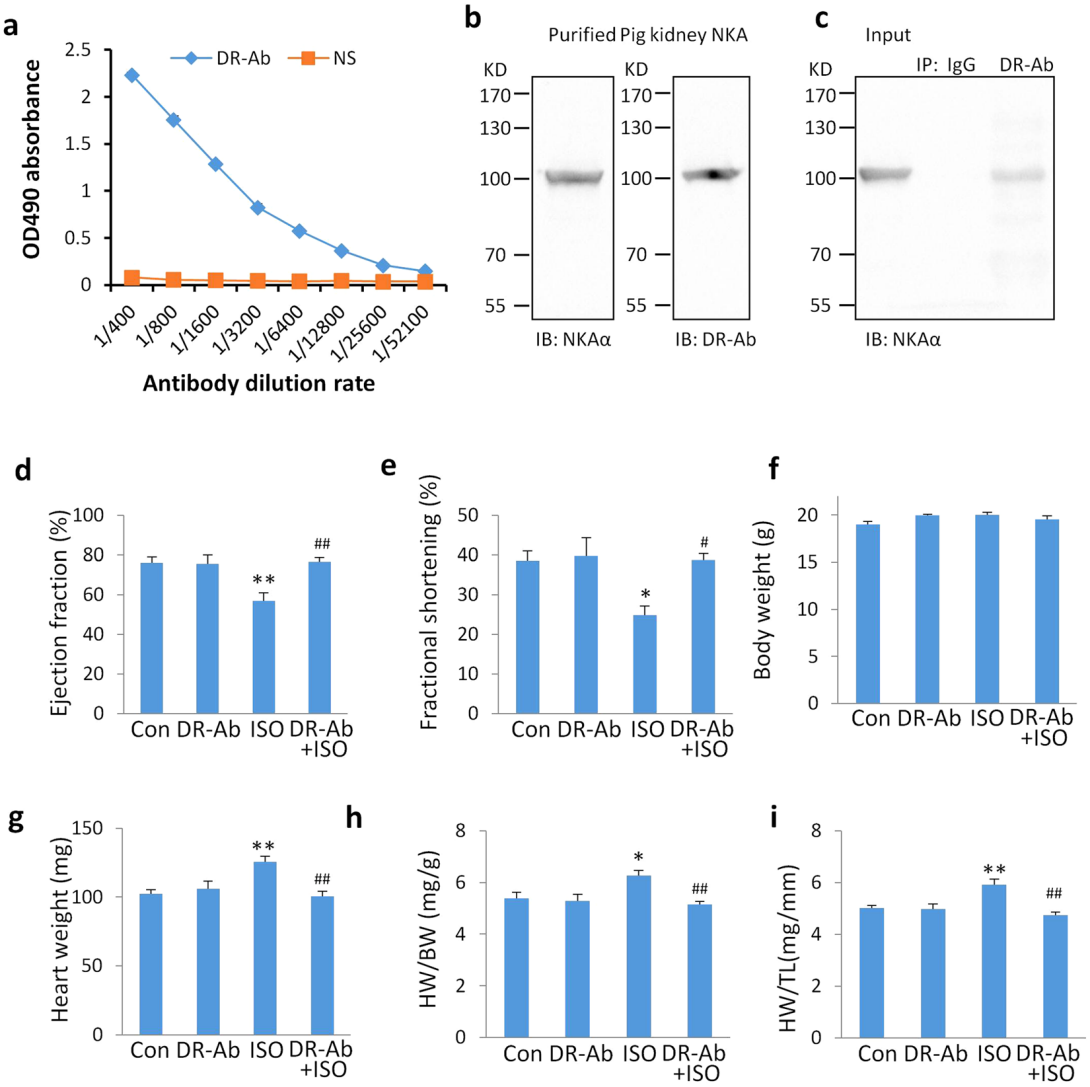
Properties of DR-Ab and its therapeutic effects in an ISO-induced mouse cardiac hypertrophy model. (**a**) Titer of DR-Ab purified from immunized rat sera and IgGs from normal rat sera (NS). (**b**) Western blots showing purified pig kidney NKA protein detected by DR-Ab and positive control (anti-NKA α, Santa Cruz, H3) at 1:1000 dilutions. IB: immunoblotting. (**c**) Immunoprecipitation assay showing that DR-Ab, but not control IgG, immunoprecipitated NKA from mouse heart lysate. IP: immunoprecipitation. (**d**,**e**) Quantitative results of ejection fraction (**d**) and fractional shortening (**e**) in each group (n = 7–8); (**f–i**) Statistical results for the body weight (**f**), heart weight (**g**), and the ratios of heart weight/body weight (HW/BW, **h**), HW/tibia length (HW/TL, i) at the end of 2 weeks post the ISO injection. n = 4–6. Mean ± SEM. *p < 0.05, **p < 0.01, ***p < 0.001 vs control; ^#^p < 0.05, ^##^p < 0.01, ^###^p < 0.001 vs ISO.

DR-Ab was purified with a protein A/G resin column and the titer was determined by ELISA against DR peptide. As show in Fig. 1a, the titer of DR-Ab was significantly higher than that of control (IgG purified from normal rat sera) at a range of 1:100 to 1:25600 dilutions. This indicates that the purified antibody was enriched with DR-Ab. After purification of DR-Ab from immunized sera, the binding of DR-Ab to NKA was detected by Western blot. As shown in Fig. 1b, Western blotting analysis with DR-Ab (1:1000 dilution) and commercial anti-Na^+^/K^+^ ATPase α (Santa Cruz, H3, 1:1000 dilution) both detected NKA protein purified from pig kidney. To further confirm the findings, immunoprecipitation experiments were performed. As shown in Fig. 1c, DR-Ab, but not control IgG, immunoprecipitated NKA from mouse heart lysate.

We then determined the protective effects of DR-Ab in the ISO-induced hypertrophy mouse model. DR-Ab (5 mg/kg) was intravenously injected 1 h before ISO challenge. M-mode echocardiography was performed to measure cardiac function 2 weeks after ISO injection. The ejection fraction (Fig. 1d) and fractional shortening (Fig. 1e), which represent the systolic function of the heart, were significantly decreased in the ISO group. These effects were attenuated by DR-Ab treatment (Fig. 1d and e).

Although no significant difference was found in the body weight (Fig. 1f), ISO significantly increased heart weight (Fig. 1g), the ratio of heart weight (HW) /body weight (BW) (Fig. 1h), and the ratio of heart weight (HW) /tibia length (TL) (Fig. 1i) when compared with those in the control group. DR-Ab pre-treatment alleviated the ISO-induced cardiac hypertrophy in the above parameters.

### DR-Ab alleviates pathological changes in the ISO-induced hypertrophy in mice

**Figure 2:**
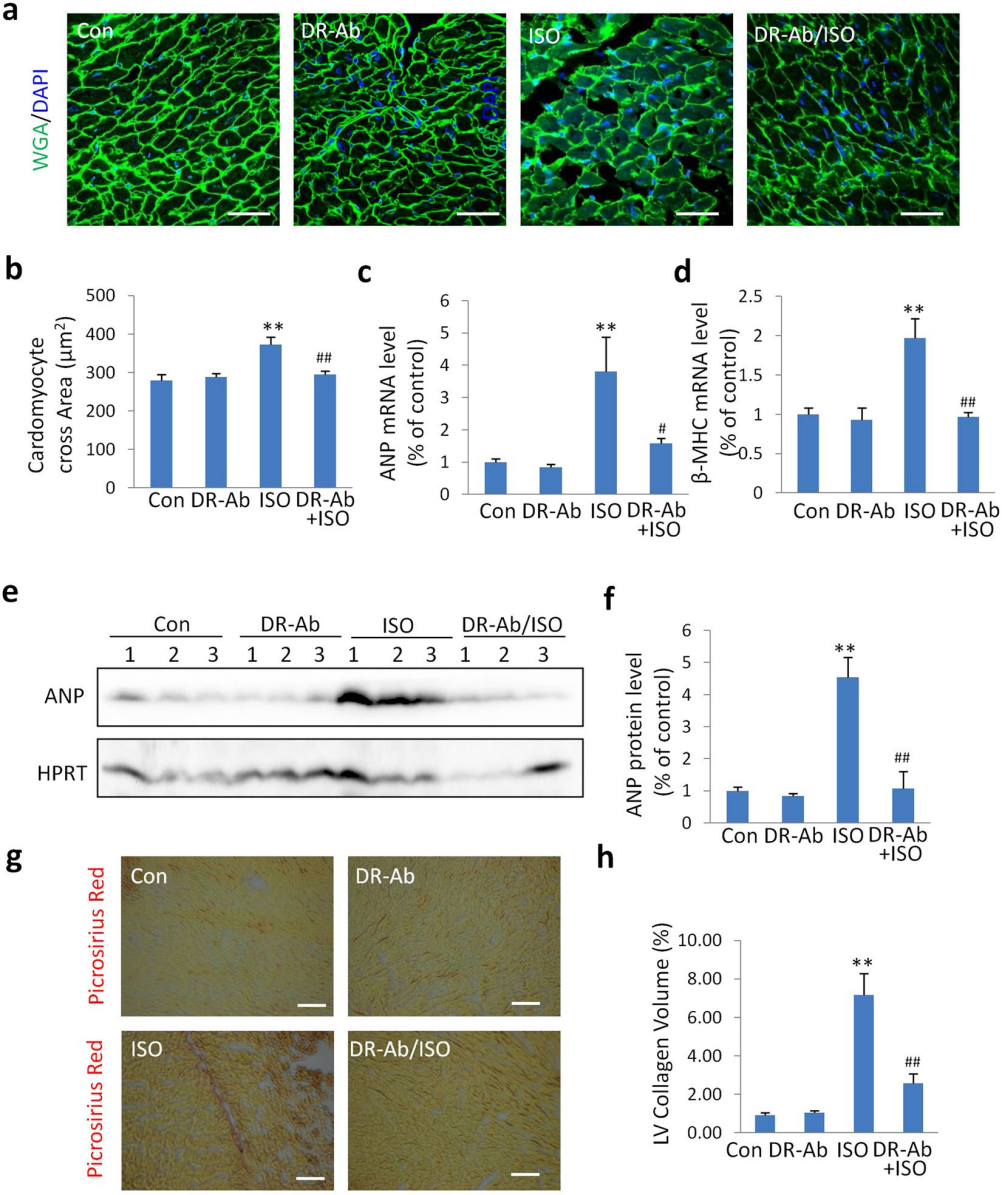
Protective effects of DR-Ab against ISO-induced hypertrophy. (**a**) Representative images of FITC-conjugated wheat germ agglutinin (WGA) staining of hearts from each group; scale bar, 50 μm; (**b**) Quantitative results of the cardiomyocyte cross sectional area in each group (n = 150 cells per group); (**c,d**) The relative mRNA levels of hypertrophic marker ANP (**c**) and β-MHC (**d**) over GAPDH (n = 4–5); (**e,f**) Representative Western blots (**e**) and quantification (**f**) of ANP to HPRT in each group (n = 5–8). (**g**) Representative images of picrosirius red staining of hearts from each group; scale bar, 50 μm; (**h**) Quantitative results of the left ventricular (LV) collagen volume in each group (n = 10 fields per group); Mean ± SEM. *p < 0.05, **p < 0.01, ***p < 0.001 vs control; ^#^p < 0.05, ^##^p < 0.01, ^###^p < 0.001 vs ISO.

To confirm the anti-hypertrophy effect of DR-Ab, we measured myocyte volume with FITC-conjugated wheat germ agglutin (WGA) staining. As shown in Fig. 2a and b, the cardiomyocyte cross-sectional area was significantly increased in the ISO group compared to the control group. However, this effect was not observed when DR-Ab was given before treatment with ISO (DR-Ab/ISO).

To further confirm the anti-hypertrophy effect of DR-Ab, atrial natriuretic peptide (ANP) and β-myosin heavy chain (β-MHC) were measured. In ISO-treated mice, the mRNA levels of ANP (Fig. 2c) and β-MHC (Fig. 2d) were increased. Western blotting analysis also demonstrated that the protein level of ANP in the ISO-treat mice was higher than the control mice (Fig. 2e and f). DR-Ab pre-treatment alleviated all these effects. Cardiac fibrosis was also determined with picrosirius red staining. As shown in Fig. 2g and h, DR-Ab significantly alleviated cardiac fibrosis in the ISO-treated mice.

### DR-Ab alleviates Isoproterenol (ISO)-induced oxidative stress

**Figure 3:**
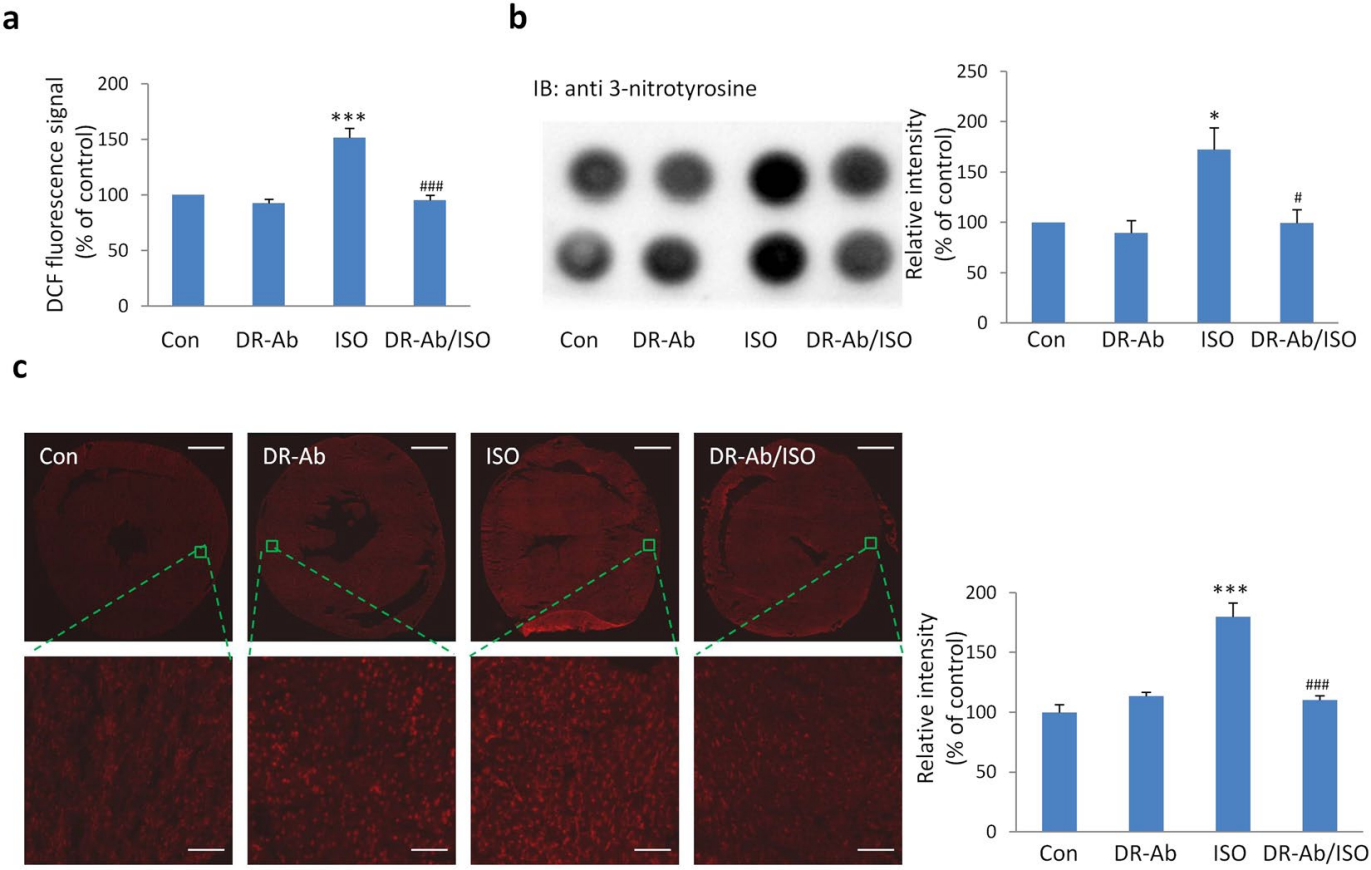
DR-Ab alleviates Isoproterenol (ISO)-induced oxidative stress. (**a**) DCF florescence of heart lysates in each group after incubation with H_2_DCF-DA for 45 min (n = 4); (**b**) Representative dot blots and quantification of total 3-nitrotyrosine levels (n = 4); (**c**) Representative images of DHE staining on the whole heart slice and enlarged view in each group. Fluorescent images of whole heart section were obtained via tile scanning with Leica DMi8 automated system. Right, quantitative results of the DHE staining in each group (3 hearts per group and 6 areas per heart). Scare bar, 1 mm for whole heart slice and 50 μm for enlarged view. Mean ± SEM. *p < 0.05, ***p < 0.001 vs control; ^#^p < 0.05, ^###^p < 0.001 vs ISO.

To investigate ISO-induced oxidative stress in the heart, we detected reactive oxygen species (ROS) with H_2_DCF-DA, total protein-bound 3-nitrotyrosine with dot blots, and superoxide with dihydroethidium (DHE). H_2_DCF-DA can diffuse easily into cells and be oxidized by ROS into 2′, 7′-dichlorofluorescein (DCF). As shown in Fig. 3a, DCF signal was significantly increased in the mouse heart lysate in the ISO group. Similarly, ISO also upregulated the levels of total protein-bound 3-NT (Fig. 3b) and superoxide (Fig. 3c). DR-Ab treatment abolished the above effects, suggesting that DR-Ab may inhibit ISO-induced oxidative stress.

### DR-Ab protects cardiomyocytes against oxidative stress-induced injury

**Figure 4:**
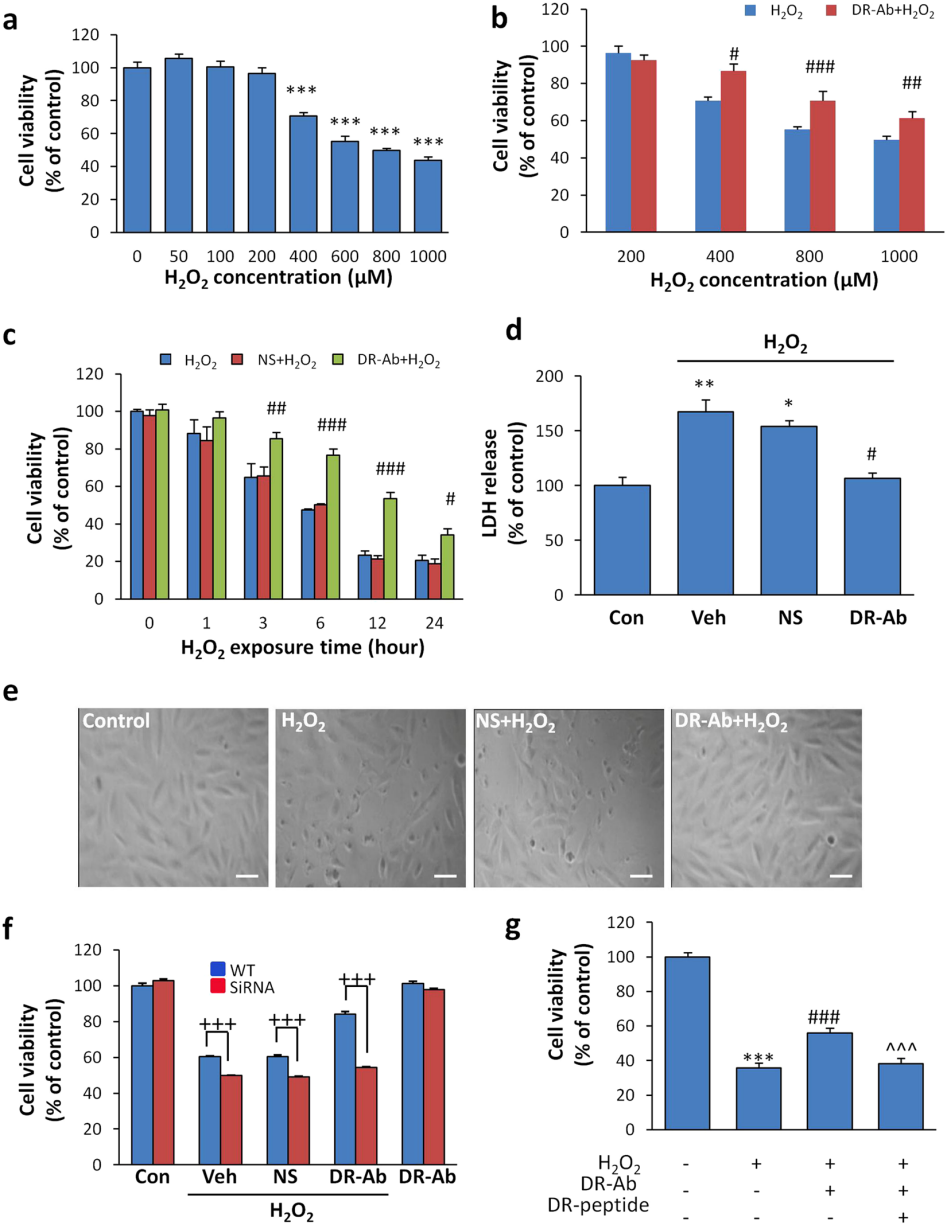
Effect of DR-Ab on ROS-induced injury in AC16 human cardiac myocytes or H9C2 rat myoblast cells. (**a**) Cytotoxicity induced by exposure to various concentrations of H_2_O_2_ for 4 hours, as measured by MTT. n = 8; (**b**) Pretreatment with DR-Ab for 1 hr inhibits cell death caused by various concentrations of H_2_O_2_. n = 8; (**c**) Time-course study showing the protective effect lasted for at least 24 hr. n = 4–8; (**d**) H_2_O_2_ increased LDH level in the culture media of H9C2 cells and DR-Ab pretreatment reduced it. n = 3. (**e**) Morphological changes (shrinking and detachment) in H_2_O_2_-treated H9C2 cells were absent in DR-Ab pretreated cells from three independent experiments; scale bar, 10 μm; (**f**) Effect of NKA α1 silencing on cell viability of AC16 cells under oxidative stress. n = 6; (**g**) Premixing DR-Ab with DR-peptide for 1 hour at room temperature under agitation abolished the protective effect of DR-Ab on AC16 cells. n = 8, Mean ± SEM. *p < 0.05, **p < 0.01, ***p < 0.001 vs control, ^#^p < 0.05, ^##^p < 0.01, ^###^p < 0.001 vs corresponding NS group. ^+++^p < 0.001 WT vs SiRNA; ^^^^^p < 0.001 vs DR-Ab group. Con: control; Veh: Vehicle (PBS); NS: Rat normal antibody, as negative control.

To study the underlying mechanisms, we observed the protective effects of DR-Ab in the AC16 human cardiomyocyte cell line. Exogenous H_2_O_2_ concentration-dependently led to cell injury in AC16 cells (Fig. 4a). Pretreatment with DR-Ab (160 μg/ml, 60 min) significantly attenuated the injury caused by 400–1000 μM of H_2_O_2_ (Fig. 4b). The concentration of H_2_O_2_ at 400 μM was therefore chosen in most of the following experiments if not otherwise indicated. The time-course analysis showed that the protective effect lasted for at least 24 hours (Fig. 4c). Antibodies purified from rat normal serum (NS) were used as a control. The necrotic cytotoxicity was measured by lactate dehydrogenase (LDH) release assay, in which LDH level was found elevated in the culture medium of H_2_O_2_-treated cells. However, incubation with DR-Ab significantly attenuated this increase (Fig. 4d). Morphological changes of the H_2_O_2_-treated cells, as characterized by shrinkage and detachment from the culture dish, were largely inhibited to a minimal level in cells incubated with DR-Ab (Fig. 4e).

To elucidate the correlation between NKA and cell vulnerability against oxidative stress, we knocked down NKA α 1 using siRNA transfection. Reduced NKA α 1 expression rendered cardiomyocytes more vulnerable to oxidative damage (Fig. 4f), and abolished the protective effect of DR-Ab, highlighting the importance of NKA in the cellular defence machinery under high ROS conditions.

To confirm the specificity of DR-Ab, we observed whether DR-peptide could abolish the protective effect of DR-Ab. As shown in Fig. 4g, pre-mixture of DR-peptide with DR-Ab for one hour completely abolished the protective effects of DR-Ab on cell viability. Taken together, these data confirm that the specific binding of DR-Ab to the DR-region on NKA is necessary to exert its protective effect.

### Anti-apoptotic effect of DR-Ab in AC16 human cardiomyocytes

**Figure 5:**
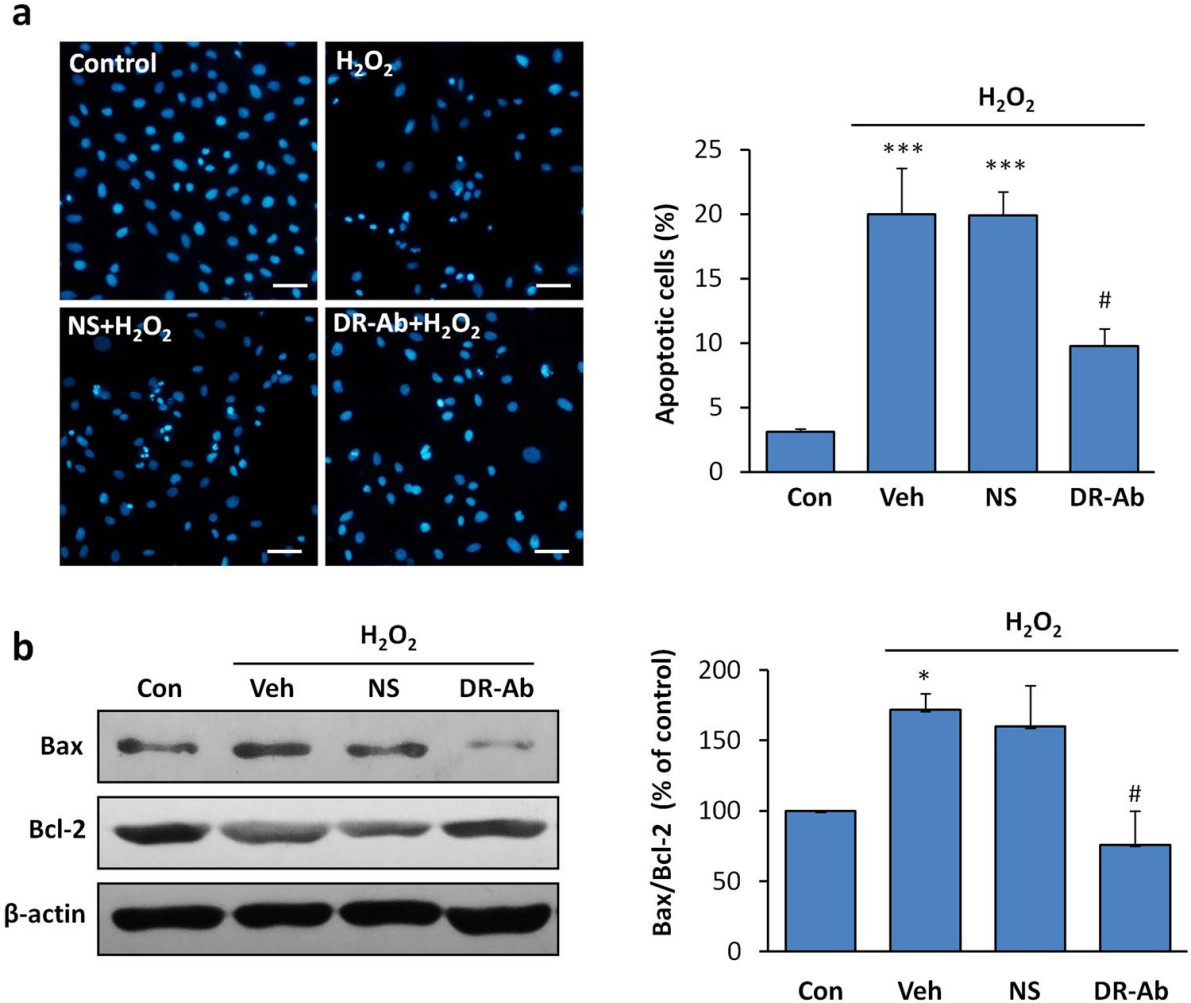
Effect of DR-Ab on H_2_O_2_-induced apoptosis in H9C2 cells. (**a**) DR-Ab reduced the number of apoptotic cells induced by overnight incubation with 100 μM H_2_O_2_, as measured by the condensed and bright nuclei in Hoechst33342 staining. From 3 independent experiments; (**b**) Representative Western blots and group analysis showing protein expression of Bcl-2, Bax. n = 4. Mean ± SEM. *p < 0.05, ***p < 0.001 vs control, ^#^p < 0.05, vs corresponding NS group. NS: Rat normal antibody, as negative control.

Next, we studied the anti-apoptotic effect of DR-Ab. AC16 cells were fixed after exposure to H_2_O_2_ (100 μM, 12 h). Cellular apoptosis was quantified using Hoechst 33342 staining. As shown in Fig. 5a, H_2_O_2_ induced apoptosis in nearly 20% of the cells, as reflected by the condensed and fragmented nuclei. In contrast, DR-Ab pretreated cells showed much reduced apoptotic activity. Western blotting analysis showed that H_2_O_2_ triggered the apoptotic pathway by down-regulating anti-apoptotic Bcl-2 protein, and up-regulating apoptotic Bax protein (Fig. 5b). Incubation with DR-Ab, but not NS, reversed H_2_O_2_ effect in the above experiments, suggesting that the protective effect was from DR-Ab.

### Membrane NKA abundance is reduced under oxidative stress via endocytosis

**Figure 6:**
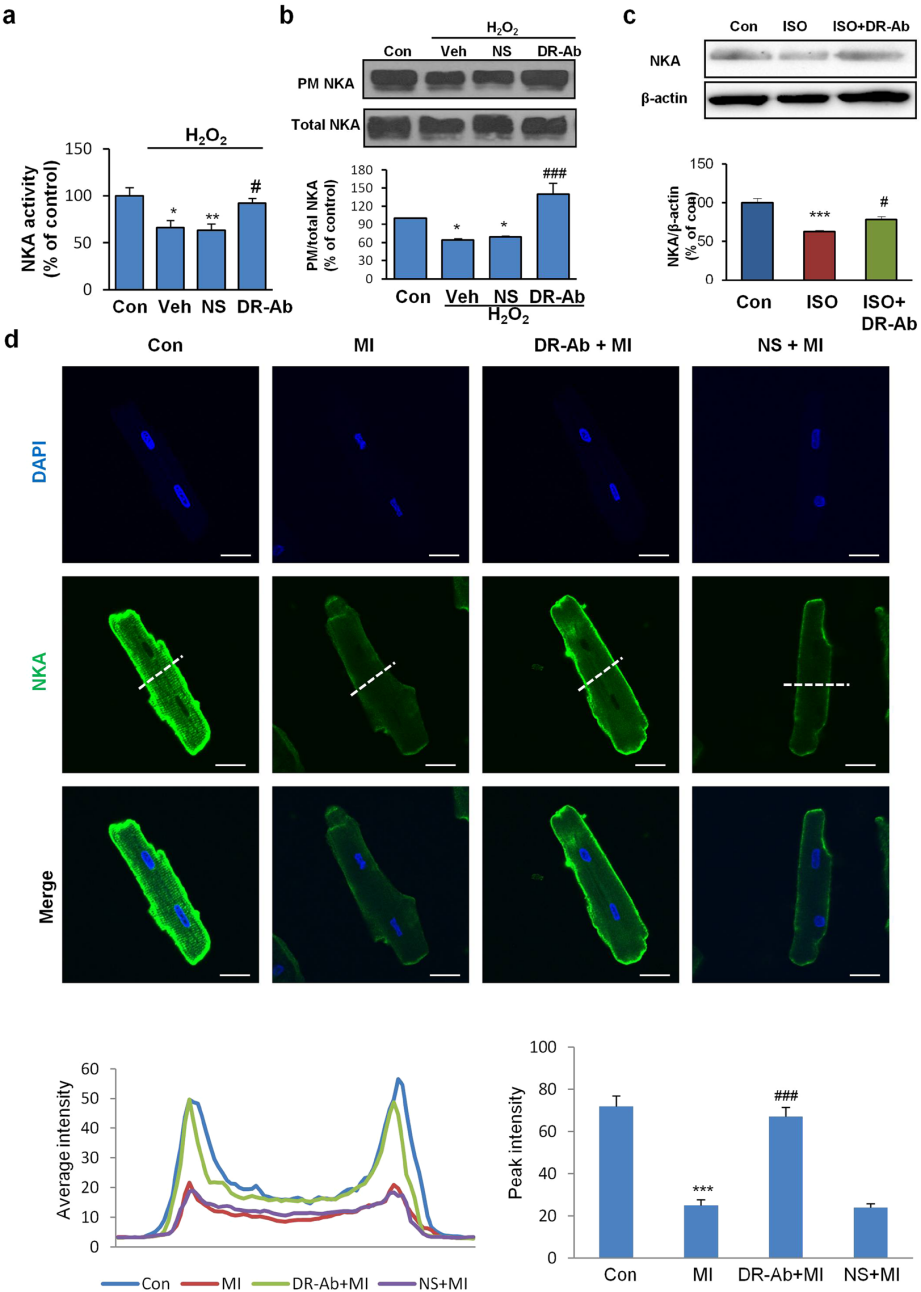
Effect of DR-Ab on the reduced NKA activity and membrane NKA abundance in H_2_O_2_ treated AC16 cells and Isoproterenol (ISO)-challenged rat hearts. (**a**) Pretreatment with DR-Ab for 1 hr rescued the inhibition of NKA activity caused by H_2_O_2_ (100 μM, 1 hr). n = 4; (**b**) Representative Western blots and group analysis showing that DR-Ab treatment reversed the plasma membrane (PM) NKA loss in AC 16 cells treated with H_2_O_2_ (100 μM, 1 hr). n = 6; (**c**) Representative Western blots and group analysis showing the effect of DR-peptide immunization on NKA abundance in the rat hearts at the end of 10 weeks post the ISO injection. n = 5; (**d**) Representative confocal immunostaining images showing reduced NKA density on the plasma membrane of isolated rat cardiomyocytes subjected to metabolic inhibition/acidosis (15 min) and normal culture medium (15 min). The lower left panel shows the fluorescent signal intensities of NKA protein collected from the lines across the cells as indicated in the green channel (NKA). The lower right histogram shows a group analysis of plasma membrane NKA staining/peak intensity. Values were the averages from 10 cells in each group. Membrane NKA abundance was maintained in cells pre-incubated with DR-Ab. Con: control, MI: metabolic inhibition/acidosis, NS: Rat normal antibody, as negative control. Scale bar, 20 μm. Mean ± SEM. *p < 0.05, **p < 0.01, ***p < 0.001 vs control; ^#^p < 0.05, vs NS/ISO.

A growing body of evidence implicates NKA as a target protein of reactive oxygen species (ROS), which modulate NKA activity and expression^22–24^. In AC16 cells, the activity of NKA appeared significantly suppressed in the presence of H_2_O_2_ but was restored to a large extent by DR-Ab treatment (Fig. 6a). In order to understand how H_2_O_2_ lowered NKA activity, we examined the effect of H_2_O_2_ on the density of NKA within the plasma membrane. Biotin-labelled cell surface NKA was reduced in response to 100 µM H_2_O_2_ for 1 hour (Fig. 6b). Cells pretreated with DR-Ab showed no such reduction (Fig. 6b). This was also demonstrated in our ISO-induced heart hypertrophy model. The NKA content in the failing heart was significantly lower than healthy controls. By contrast, animals with DR-Ab exhibited an upward trend in the amount of NKA (Fig. 6c). Membrane NKA loss was also found in isolated rat cardiomyocytes that were subjected to metabolic inhibition/acidosis (15 minutes), followed by incubation with culture medium for 15 minutes. The decrease in cell surface density of NKA was also inhibited by incubation with DR-Ab (Fig. 6d). The membrane NKA signals were quantified with ImageJ as shown in the *right panel*.

**Figure 7:**
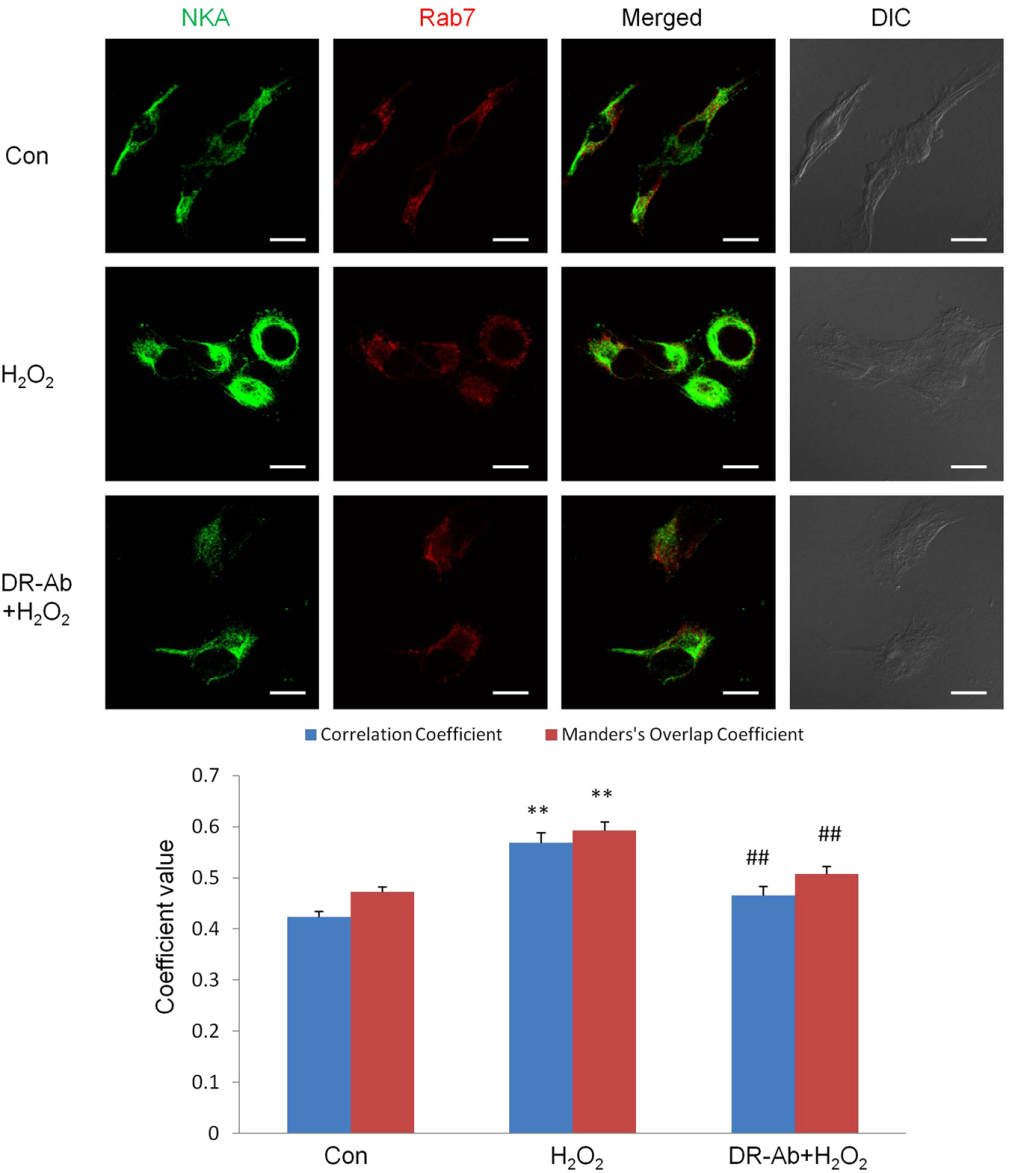
Immunofluorescence staining showing the endocytosis of NKA in AC16 cells. Representative immunofluorescence staining and co-localization analysis showing the increased co-localization of NKA (Green) with late endosome marker, rab7 (Red) in AC16 cells treated with 100 µM H_2_O_2_ for 1 hr. n = 17. Scale bar, 10 μm.

**Figure 8:**
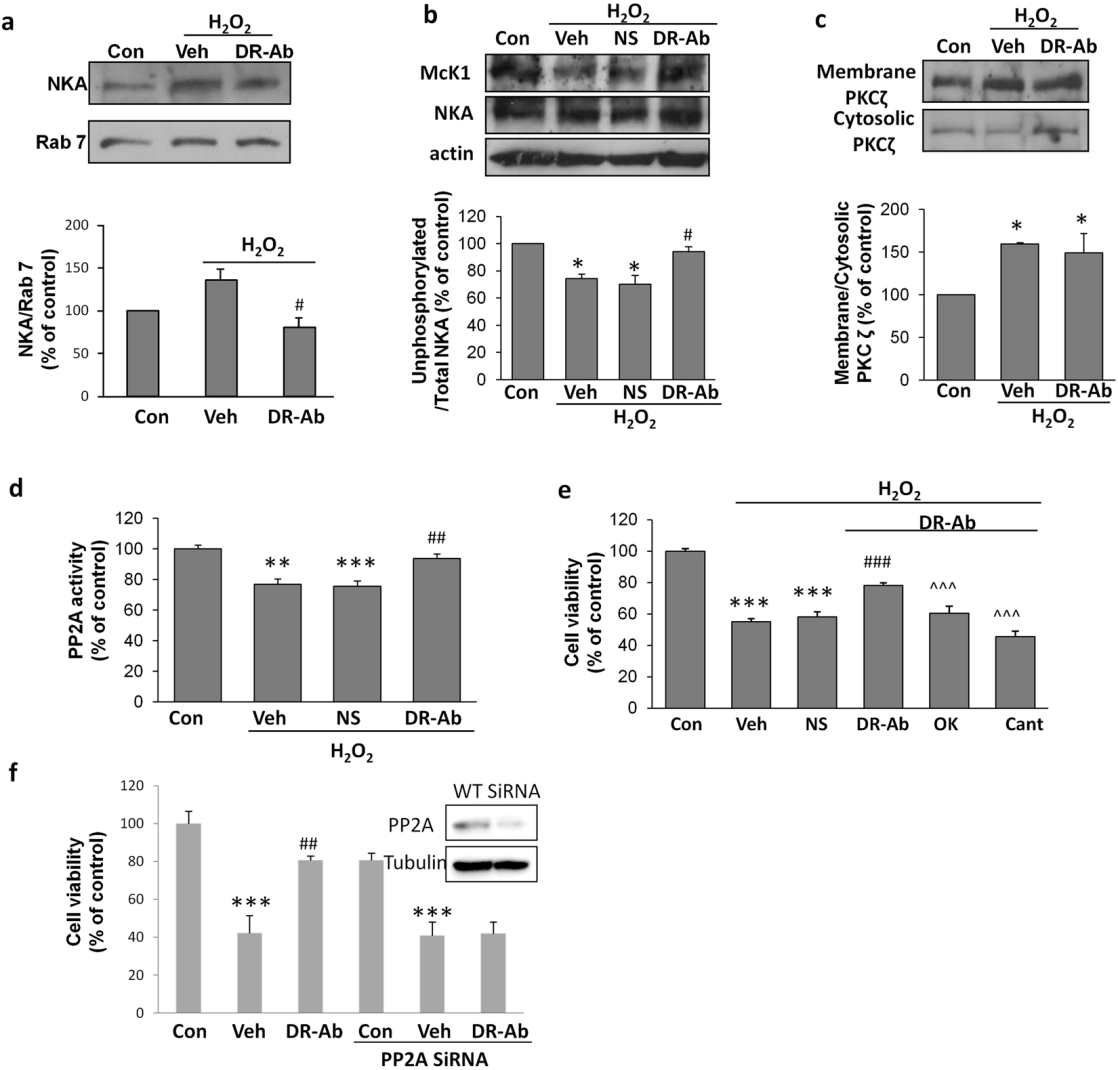
Involvement of PP2A in H_2_O_2_-induced NKA endocytosis and cardioprotective effect of DR-Ab. (**a**) Representative Western blots and group analysis showing NKA in endosomes isolated from cells of different treatment groups. Incubation with 100 µM H_2_O_2_ for 1 hr induced enrichment of NKA in the endosomes. n = 6; (**b**) Representative Western blots and group analysis of McK1 showing the unphosphorylated NKA at Ser18 in H9C2 rat myoblast cell line exposed to 100 µM H_2_O_2_ for 30 min. n = 3; (**c**) Representative Western blots and group analysis of PKCζ in the membrane and cytosolic fractions of cell lysate. n = 4; (**d**) Effect of H_2_O_2_ (100 µM, 30 min) and DR-Ab (160 ug/ml, 1 h pretreatment) on PP2A activity in AC16 cells. n = 3; (**e**) Effect of PP2A inhibitors, okadaic acid (OK, 100 nM) and cantharidin (Cant, 1 µM), on the DR-Ab conferred antioxidative protection. n = 8. (**f**) Effect of PP2A silencing on cell viability of AC16 cells under oxidative stress. Insert, Western blots showing protein expression of PP2A. n = 8. Mean ± SEM. *p < 0.05, **p < 0.01, ***p < 0.001 vs control, ^#^p < 0.05, ^##^p < 0.01, ^###^p < 0.001 vs H_2_O_2_ treatment group, ^^^^^p < 0.001 vs DR-Ab + H_2_O_2_ group. Veh: PBS. NS: Normal rat antibody.

To define the mechanism underlying the loss of membrane NKA, the colocalization of NKA with endosomes was studied by immunoflurorescence. As shown in Fig. 7, the co-localization of NKA and rab7, a late endosome marker, was increased upon H_2_O_2_ treatment. Treatment with DR-Ab reversed the translocation of membrane NKA to endosomes. These data were further confirmed with Western blotting analysis (Fig. 8a), where endosomes isolated from AC16 cells showed enriched NKA content after 1 hour exposure to 100 μM H_2_O_2_, and DR-Ab treatment reversed the NKA enrichment in endosomes. Our data indicate that DR-Ab may prevent NKA endocytosis during oxidative stress.

### The cardioprotective effect of DR-Ab is mediated by activation of PP2A and subsequent stabilization of functional NKA

Several reports have documented the phosphorylation of certain serine residues on NKA α subunit as an essential event that triggers the endocytosis of the sodium pump^25–28^. Here we studied the level of phosphorylated Ser18 using a McK1 antibody, which recognizes the unphosphorylated α1 subunit (Ser18). As shown in Fig. 8b, H_2_O_2_ induced phosphorylation of Ser18, which was reversed by DR-Ab. It was reported that PKCζ and PP2A are responsible for the phosphorylation state of NKA^16,21,29–31^. To understand whether the reduction of phosphorylation by DR-Ab was a result of inhibited kinase or enhanced phosphatase, we first examined the activity of PKCζ in H_2_O_2_-treated AC16 cells. Increased PKCζ translocation from the cytosol to the membrane fraction occurred 30 minutes after H_2_O_2_ treatment and DR-Ab failed to have any impact on PKCζ activation (Fig. 8c). On the other hand, DR-Ab mitigated the H_2_O_2_ suppression on PP2A activity (Fig. 8d), suggesting the involvement of PP2A in the regulation of NKA Ser18 phosphorylation by DR-Ab.

To test if activation of PP2A by DR-Ab underlies the protective effect, we blocked PP2A using its specific inhibitors, okadaic acid (100 nM) and cantharidin (1 µM). As shown in Fig. 8e, the protective effect induced by DR-Ab against H_2_O_2_ was abolished with the application of okadaic acid or cantharidin. Similarly, specific knockdown of PP2A with siRNA also abolished the protective effects of DR-Ab on cell viability (Fig. 8f). These data confirm that the cardioprotective effect of DR-Ab was mediated by PP2A.

## Discussion

As one of the first ion pumps discovered, the active transport functions of NKA has been well studied^32^ and implicated in the pharmacological management of heart diseases, as in the case of digitalis glycosides, which inhibit NKA to modulate heart contractility. Over the last few decades, another function of the NKA as a signal transducer has emerged with an increasing body of evidence^33,34^. Our group and others have shown that the activation of the NKA-associated signaling pathways led to enhanced heart contractility and cardioprotection^12,14,35^, suggesting novel approaches to devise treatment strategy for heart conditions. Despite the advance in our knowledge of NKA in cardiac diseases, a clear definition of the role NKA plays in maintaining viable myocardium is still lacking. In this study, we demonstrated that NKA expression and function are impaired by oxidative stress via endocytosis, and that targeting the DR region on NKA α-subunit with the DR-Ab activates PP2A and stabilizes cell surface NKA from internalization, which may translate into its long-term preservation in failing hearts. Moreover, DR-Ab alleviates oxidative damage in cardiomyocytes and myocardium.

A wealth of studies have implicated the involvement of ROS in various physiological and pathological events in the cardiovascular system. ROS participate in redox signalling in normal healthy cardiomyocytes, and dysregulated ROS pose oxidative challenge to viable myocardium, which underlies the development of a series of cardiac diseases such as ischemia/reperfusion injury, myocardial infarction, cardiac hypertrophy, and heart failure^36–38^. Given the pivotal role of ROS in the development of cardiac conditions, we studied the mechanisms underlying the cardioprotective effect of DR-Ab with the H_2_O_2_-induced oxidative stress model.

We also studied the cardioprotective effects of DR-Ab in hypertrophy. Sustained elevation of plasma and interstitial catecholamine and ROS generation are characteristics in pathologic heart conditions, particularly heart failure. Administration of excessive amount of catecholamines, such as ISO, results in massive myocardial damage and extensive fibrosis^39^, and a subsequent infarct-like myocardial necrosis^40^ with reduced functional capacity of the heart^41,42^. ISO-treated rats showed necrotic myocardium death and progressive enlargement of the LV cavity out of proportion to mass, similar to patients with discrete myocardial infarction. For this reason, we evaluated the effect of DR-Ab on ISO-induced hypertrophy model. DR-Ab attenuated the enlargement of the LV and reduced ventricular mass, providing additional evidence that DR-Ab prevents adverse remodelling of myocardium. Taken together, the above data clearly suggest that DR-Ab may protect cardiomyocytes against cellular injury under oxidative conditions.

The presence and abundance of plasma membrane proteins is finely regulated by a balance between anterograde and retrograde vesicular trafficking. The disruption of such balance often results in deleterious cellular physiological consequences. In this study, we found PKCζ was activated, while PP2A inhibited under oxidative stress, which are in line with previous reports^43,44^. Together, they account for NKA’s reduction on cell surface and enrichment in endosomes when cells were treated with H_2_O_2_. Eventually, the unbalanced NKA trafficking gives rise to reduced NKA level in unhealthy myocardium, as described in our ISO-induced mouse heart failure and by others^7–11^. Reduction of NKA renders cells more vulnerable to high ROS environments commonly seen in cardiac pathological conditions. Incubation with DR-Ab failed to affect PKCζ activation, but restored the suppressed PP2A activity, suggesting PP2A underlies the anti-endocytosis effect of DR-Ab. Modulation of PP2A activity by DR-Ab might be achieved by direct interaction between NKA and PP2A^16,45^. Upon binding to NKA, it is speculated that DR-Ab may induce a transformational change leading to increased affinity of PP2A to the first 90 amino acid in the NKA α-subunit^16^. These data support our findings in cellular models and implicate the possible long-term consequence of up-regulated NKA endocytosis under high ROS environment. Moreover, the present *in vivo* evidence suggests that the DR-Ab’s ability in stabilizing NKA from endocytosis may translate into higher NKA concentration in failing hearts, which may be the underlying mechanism for the beneficial effects of DR-Ab.

Combined with our previous findings that activation of NKA produces cardioprotection in isolated hearts through the NKA signalling complex^12^, it is reasonable to postulate that by stabilizing the content of functional NKA under oxidative stress, DR-Ab is able to prevent the loss of NKA activity both as an ion pump and signal transducer, therefore alleviating oxidative injury in cardiac pathologies. However, given the ubiquitous expression of the NKA, there may be some concern as to whether systemic NKA manipulation can cause over-stimulation of NKA in cardiac and other tissues. However, we have yet to observe any over-expression of NKA on plasma membrane following DR-Ab treatment. In addition, our preliminary data also demonstrated that overexpression of exogenous NKA reduces endogenous NKA generation and keeps the total NKA at the normal level. Moreover, we did not observe any significant changes in heart function or structure in DR-Ab-treated mice or DR-peptide-immunized animals.

Although more work needs to be done to fully elucidate the protective effect of DR-Ab in heart, the current findings provide new mechanistic insight of the role DR-Ab plays under oxidative stress. While ongoing research has been devoted to the exploration of new targets to treat heart diseases caused by loss of viable cardiomyocytes, NKA, an old enzyme, has emerged as a key player that deserves more attention in cardioprotection. The current study has shed light onto functions of NKA during oxidative stress and how its preservation may grant us novel approaches to fight cardiovascular diseases.

## Material and Methods

### Animals

All animal experimental procedures in this study were approved by the Institutional Animal Care and Use Committee of the National University of Singapore. All methods were performed in accordance with the relevant guidelines and regulations.

### Chemicals and reagents

PP2A and rab7 antibodies were purchased from Cell Signaling (Danvers, USA). NKA α antibody, HPRT antibody, ANP antibody, 3-nitrotyrosine antibody, tubulin antibody, goat anti-rabbit and goat anti-mouse secondary antibodies were purchased from Santa Cruz Biotechnology (Santa Cruz, USA). NKA α1 antibody (464.6, ab7671) was purchased from Abcam (Cambridge, UK). Alexa Fluor 568 conjugated goat anti-rabbit IgG (H + L), Alexa Fluor 488 conjugated goat anti-mouse IgG (H + L), FITC-conjugated wheat germ agglutinin were from Invitrogen Corporation (Carlsbad, USA). The McK1 antibody was a generous gift from Dr. K. Sweadner (Massachusetts General Hospital, MA). SensoLyte® FDP Protein phosphatase assay kit was from AnaSpec, Inc (AnaSpec, Inc, Japan). Primers for qPCR were from Integrated DNA Technologies. Collagenase (type I) was from Worthington Biochemical. DMEM medium, DMEM/F12 medium and fetal bovine serum were from HyClone. All other reagents were purchased from Sigma Chemical Company (St. Louis, USA).

### The generation of a DR-region-specific antibody

Adult Sprague-Dawley male rats (6–8 weeks) were immunized subcutaneously with KLH (Keyhole Limpet Hemocyanin) conjugated DR region peptide (^897^DVEDSYGQQWTYEQR^911^) bi-weekly for a total of four injections. The initial dose was 200 µg protein emulsified with CFA (complete Freund’s adjuvant) per rat followed by 100 µg protein emulsified with IFA (incomplete Freund’s adjuvant) per rat every other week. The immunization generally took 8 weeks (4 injections in total), but in some cases were extended in animals with low antibody production. 5 days after the last immunization, rats were euthanized and exanguinated by cardiopuncture. The blood was incubated at 37 °C for 30 min, and then centrifuged at 5000 *g* for 20 min. The sera were stored at −80 °C until use. In some experiments, the rats were immunized with DR-peptide following the above protocol, and the effect of circulating DR-Ab was studied in these animals.

For antibody purification, the Protein A/G resin column was first balanced by ten times volume of phosphate buffer solution (PBS, pH 7.2), and then the immune sera were passed through the column. The non-specific proteins were washed away with washing buffer. Antibodies were eluted with 0.1 M glycine (pH 3.0) and the eluted fractions were immediately adjusted to physiologic pH by adding 100 μl of the 1 M phosphate (pH 8.0) to 1 ml of eluate. The elution was monitored by measuring the absorbance at 280 nm or by protein assay such as BCA™ Protein Assay Kit. The protein content of the purified fractions containing the antibody was determined by BCA™ Protein Assay Kit.

Antibody titer was measured with typical ELISA procedure. In brief, DR region peptide diluted in sodium carbonate buffer was loaded into the ELISA plate and incubated at 4 °C overnight. The plate was washed with PBS-T buffer and blocked with 3% BSA/PBS-T. Serial two-fold dilutions of purifed DR antibody or IgGs purified from normal rat sera (NS) were prepared and incubated in the plate for 3 hour at room temperature. The plate was wash with PBS-T and incubated with horseradish peroxidase-conjugated anti-rat IgG at room temperature for 1 h and washed three times in PBS-T. 3′, 3′, 5′, 5′-Tetramethylbenzidine (TMB) was added and incubated at room temperature for 30 min. The reaction was stopped by adding 1 M H_2_SO_4_. Absorbance at 450 nm was recorded with plate reader.

### Isolation of ventricular myocytes

Ventricular myocytes were isolated from adult Sprague-Dawley rats, using standard enzymatic methods. Briefly, a central thoracotomy was performed after Sprague-Dawely rats (200~250 g, male) were anesthetized with Ketamine/xylazine (75/10 mg/kg) mixture and administrated of heparin (1000 IU) by intraperitoneal injection. The heart was quickly excised and perfused on a Langendorff system with calcium-free Tyrode’s solution (in mM): 137 NaCl, 5.4 KCl, 1 MgCl_2_, 10 HEPES, 10 Glucose, pH 7.4 at 37 °C. After 5 min, the perfusate was changed to the Tyrode’s solution containing 1 mg/ml collagenase (type I) and 0.28 mg/ml protease (type XIV) and perfused for 30 min. The left ventricular tissue was gently minced, filtered, and washed three times in calcium-containing Tyrode’s solution. The cells were allowed to stabilize at room temperature for 30 min. The isolated ventricular myocytes were pretreated with DR Ab or vehicle (PBS) for 1 h, and then subjected to ischemic buffer (glucose-free Krebs buffer containing 5 mM sodium lactate, 20 mM 2-deoxy-d-glucose (an inhibitor of glycolysis), 20 mM sodium dithionite (Na_2_S_2_O_4_, an oxygen scavenger), pH 6.6) for 15 minutes and replaced with DMEM medium for 15 minutes. The cells were then processed for immunofluorescence labelling.

### Purification of NKA

NKA was purified from the outer medulla of pig kidney by differential centrifugation and SDS-treatment with modification. Briefly, medulla were homogenized in homogenizing medium (25 mM imidazole, 250 mM sucrose, 1 mM EDTA, pH7.4), then centrifuged at 3700 g for 20 min at 4 °C. The supernatant was centrifuged at 7400 g for 20 min at 4 °C and the resultant supernatant was centrifuged at 38 000 g for 40 min at 4 °C. The pellet-containing microsomal fraction was suspended in the homogenizing medium and incubated with 0.1% SDS at room temperature overnight. The resultant suspension was centrifuged at 127 000 g for 50 min at 10 °C. The pellet was resuspended in washing buffer (20 mM histidine, 250 mM sucrose, 0.9 mM EDTA, pH7.0). After three rounds of centrifugation/wash, the final pellet was resuspended in the homogenizing medium to a protein concentration of 1 mg/mL. The aliquots were stored at −80 °C.

### Isoproterenol (ISO)-induced heart failure in mice

Animals were randomly assigned to various treatment groups. For DR-Ab groups, animals were pretreated with intravenous DR-Ab (5 mg/kg) 1 h before ISO injection. Cardiac hypertrophy was induced by subcutaneous injection of ISO (150 mg/kg) daily for five days. The animals were raised for another 2 weeks before humanely killed. The somatic and organ weights were measured to evaluate the ISO-induced heart hypertrophy. Heart tissues were collected for biochemistry studies. To study the involvement of oxidative stress in ISO-induced heart failure model, some animals were euthanized after last injection of ISO and the heart tissues were collected for oxidative stress measurements.

### Dihydroethidium staining

Dihydroethidium (DHE), a lipophilic cell-permeable dye, can undergo oxidation and the product binds to the double-stranded DNA, causing amplification of a red fluorescent signal, indicating ROS production. Mouse hearts were collected and placed in optimum cutting temperature (OCT) formulation (Sakura, USA). The hearts were immediately frozen on dry ice. The hearts were sectioned (10 μm; −22 °C) using a Leica cryostat and placed on slides before being incubated with DHE (10 μM) in PBS in a light-protected, humidified chamber for 30 min at 37 °C. Fluorescent images (excitation 488 nm and emission 574–595 nm) of whole heart section were obtained via tile scanning with Leica DMi8 automated system.

### Wheat germ agglutinin (WGA) staining

To determine the myocyte cross-sectional area, heart sections were stained with FITC-conjugated wheat germ agglutinin (WGA, Invitrogen Corp) to visualize the membranes and with DAPI to observe the nuclei. More than 150 myocytes were examined in each group. Fluorescent images were taken with Olympus FV1000 confocal laser scanning microscopy and analyzed with Image J.

### Picrosirius red staining

To evaluate cardiac fibrosis, heart sections were stained with Picrosirius red. The slides were examined with Olympus BX50 microscope and analyzed with Image J.

### H_2_DCF-DA fluorometric assay

The relative levels of ROS generated in the hearts were also monitored by a fluorometric assay using H_2_DCF-DA (Sigma). The hearts were removed, quickly frozen, and homogenized in 40 mM ice-cold Tris·HCl and 0.1% Tween buffer, pH 7.4. The homogenate was divided into two equal fractions, loaded with 5 μM H_2_DCF-DA or solvent DMSO. All samples were incubated for 45 min at 37 °C, and then fluorescence (excitation 504 nm and emission 529 nm) was measured with TECAN SPARK 10 M.

### Dot blot analysis

Heart total protein extract samples (5 µl, 250 ng), 12% SDS (5 µl), and 5 µl modified Laemmli buffer containing 0.125 M Tris base, pH 6.8, 4% (v/v) SDS, and 20% (v/v) glycerol were incubated for 20 min at room temperature and then loaded onto nitrocellulose membrane. The membrane was blocked in blocking buffer (3% bovine serum albumin) in PBS-T for 1 h and incubated with an anti- 3-NT antibody in PBS-T for 90 min. The membrane was washed in PBS following primary antibody incubation three times at intervals of 5 min each. The membrane was incubated with a horseradish peroxidise-conjugated anti-mouse IgG for 1 h. The membrane was washed three times in PBS for 5 min each and developed with chemiluminescence kit (GE Healthcare). Images were acquired with Chemi-Doc MP (Bio-Rad, USA) and analysized with Image J.

### qPCR

Total RNA from heart tissue was isolated with TRIzol reagent (Invitrogen Life Technologies), and 1 μg of RNA was used for the reverse transcription reaction with RevertAid First Strand cDNA synthesis kit (Thermo Scientific). Quantification of mRNA level was done with the GoTaq@qPCR kit (Promega). The PCR primers used in this study were: Glyceraldehyde 3-phosphate dehydrogenase (GAPDH), forward 5′-ACTGAGCAAGAGAGGCCCTA, reverse TATGGGGGTCTGGGATGG AA; Atrial natriuretic peptide (ANP), forward GCCCTGAGTGAG CAGACTG, reverse GGAAGCTGTTGCAGCCTA; beta-myosin heavy chain (β-MHC), forward ACCTACCA GACAGAGGAAGA, reverse TTGCAAAGAGTCCAGGTCTGAG.

### Echocardiography

We studied the mouse cardiac function using a high-resolution echocardiography system (Vevo 2100, VisualSonics). Briefly, mice were anesthetized with isoflurane inhalation. The heart rate was maintained at around 400 BPM (beats per minute) and body temperature was maintained at 37 °C by placing the animal on a heating pad. With a 40 MHz probe on short axis M-mode images, we measured the thickness of interventricular septum, LV posterior wall and LV internal diameter at diastolic and systolic states. Next, ejection fraction and fractional shortening were calculated.

### Cell culture and treatment

AC16 human cardiomyocyte cell line was purchased from Land Biology (Guangzhou, China). Cells were maintained in DMEM/F12 medium supplemented with 12.5% FBS and 1% penicillin-streptomycin. This basic medium was replenished every 3 days. H9C2 rat cardiomyoblast cell line was purchased from the American Type Culture Collection (ATCC; Manassas, VA, USA) and maintained in DMEM medium supplemented with 10% FBS and 1% penicillin-streptomycin. In most experiments, cells were pretreated with DR-Ab for 60 min and then incubated in freshly prepared medium containing 400 μM H_2_O_2_ for 4 h. These cells were then used to measure cell viability, apoptosis, PP2A activity, and NKA activity.

### Cell viability and apoptosis assay

Cell viability was evaluated with the 3-(4,5-dimethylthiazol-2-yl)-2,5-diphenyltetrazolium bromide (MTT) method as described previously with modification^46^ and cell counting kit-8 according to manufacturer’s instruction. To visualize nuclear morphology, cells were fixed in 4% paraformaldehyde and stained with 2.5 μg/ml Hoechst33342 DNA dye. Uniformly stained nuclei were scored as healthy viable cells. Condensed or fragmented nuclei were scored as apoptotic. To obtain unbiased counting, Petri dishes were coded, and cells were scored blindly without knowledge of their prior treatment.

### Immunofluorescence labelling and protein co-localization

Isolated adult rat cardiomyocytes and AC16 cells were fixed in 4% paraformaldehyde and then permeabilized with 0.1% Saponin, followed by incubation with mouse anti-NKA antibody and rabbit anti-rab7 antibody overnight at 4 °C. After washing three times with PBST (PBS that contained 0.1% Tween-20), cells were incubated with goat anti-mouse-Alexa 488 or goat anti-rabbit-Alexa 568 for 1 hr at room temperature before mounting with DAPI-containing mounting medium (Invitrogen, Carlsbad, CA, USA). Photos were taken using a confocal fluorescence microscope (Nikon or Olympus). Following acquisition, images were analyzed with the Image J (NIH). The correlation coefficient and the Mander’s overlap coefficient between the respective channels were quantified with the colocalization plug-in of the Image J software.

### PP2A activity and NKA activity assay

PP2A and NKA activities were determined using a Fluorimetric Senso Lyte FDP Protein Phosphatase Assay Kit (AnaSpec, Japan) according to manufacturer’s instructions.

### Biotinylation of cell surface proteins

Surface proteins were labelled with EZ-Link NHS-SS-biotin (1 mg/ml, Pierce Chemical Co., Rockford, IL, USA) for 1 h as described before^16^. Cells were then rinsed with PBS containing 100 mM glycine thoroughly to quench unreacted biotin and then lysed in modified radio-immuno-precipitation assay (RIPA) buffer (50 mM Tris-HCl, pH 8; 150 mM NaCl; 1% Nonidet P-40 and 1% sodium deoxycholate; 10 μg/ml leupeptin; 100 μg/ml TPCK; and 1 mM PMSF). Proteins (150–300 μg) were incubated overnight at 4 °C with end-over-end shaking in the presence of Streptavidin beads (Pierce Chemical Co.). Beads were thoroughly washed, resuspended in 30 μl loading buffer, and analyzed by Western blots.

### Preparation of endosomes

Endosomes were fractioned on a floatation gradient using the technique previously described^25,26,47^ with some modifications. Briefly, cells were washed with ice-cold PBS and homogenization buffer containing 250 mM sucrose and 3 mM imidazole, pH7.4. The cells were gently homogenized using a Dounce homogenizer with homogenization buffer supplemented with protease inhibitors, followed by centrifugation at 4 °C, 2000 × *g* for 10 minutes. The supernatant was adjusted to 40.6% sucrose and was loaded at the bottom of a centrifuge tube. A 35% sucrose solution with 3 mM imidazole and 0.5 mM EDTA and homogenization buffer was added in sequence. The samples were centrifuged for 1.5 hours at 210,000 × *g* in a Beckman Ti90 rotor. Endosomes were collected at the homogenization buffer-35% sucrose interface. Immunoblots for rab7 were carried out to identify the endosome fraction.

### NKA α1 and PP2A C knockdown by siRNA transfection

RNA interference was used to silence the expression of NKA α1and PP2A C in AC16 cells. Cells were plated in six-well plates and cultured in antibiotic-free medium overnight before they were transfected with siRNAs (Santa Cruz Biotechnology, CA, USA) using Lipofectamine 2000 according to the manufacturer’s instructions. Cells were cultured for at least 3 days before various assays were carried out.

### Western blotting analysis

Protein concentrations were determined by the Lowry method. Protein samples were separated by 8–12% SDS-PAGE and transferred onto a nitrocellulose membrane. After blocking at room temperature in 10% milk in TBST buffer (10 mM Tris-HCl, 120 mM NaCl, and 0.1% Tween 20, pH7.4) for 1 h, the membrane was probed with various primary antibodies at 4 °C overnight. Membranes were then washed three times in TBST, followed by incubation with 1:10000 dilutions of horseradish peroxidise-conjugated anti-rabbit/mouse IgG at room temperature for 1 h and washed three times in TBST. Visualization was carried out using an enhanced chemiluminescence kit (GE Healthcare). The density of the bands was quantified by densitometry analysis of the scanned blots using ImageJ.

### Immunoprecipitation

Briefly, mouse heart lysate (50 μg) was incubated with control IgG or DR-Ab (1 μg) overnight at 4 °C. Then protein A/G conjugated beads (100 μl) were added and incubated for 4 h at 4 °C. The beads were washed three times and eluted with SDS sample buffer (40 ul). The eluted samples (20 ul) were subjected to Western blot analysis.

### Statistical analysis

Values are presented as Means ± SEM. One-way or two-way analysis of variance followed by Bonferroni Significant Difference test was used to analyze the differences within groups where appropriate. Significance level was set at p < 0.05.

## Electronic supplementary material

Supplementary Information

## Data Availability

All data generated during this study are included in this published article (and its Supplementary Information files). The data can be also obtained from the authors on reasonable request.
