# Supplementary material for: DR region of Na+-K+-ATPase is a new target to protect heart against oxidative injury
Source: Sci Rep. 2018 Aug 30;8:13100. doi: 10.1038/s41598-018-31460-z (PMC6117330; doi:10.1038/s41598-018-31460-z)

DR region of Na^+-K^+-ATPase is a new target to protect heart against oxidative injury

Fei Hua, Zhiyuan Wu, Xiaofei Yan, Jin Zheng, Haijian Sun,
Xu Cao, Jin-Song Bian

Fig 1b

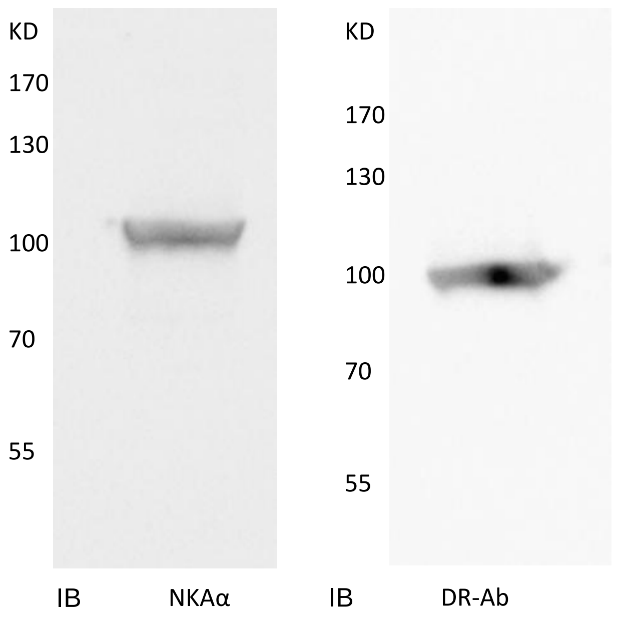

Fig 1c

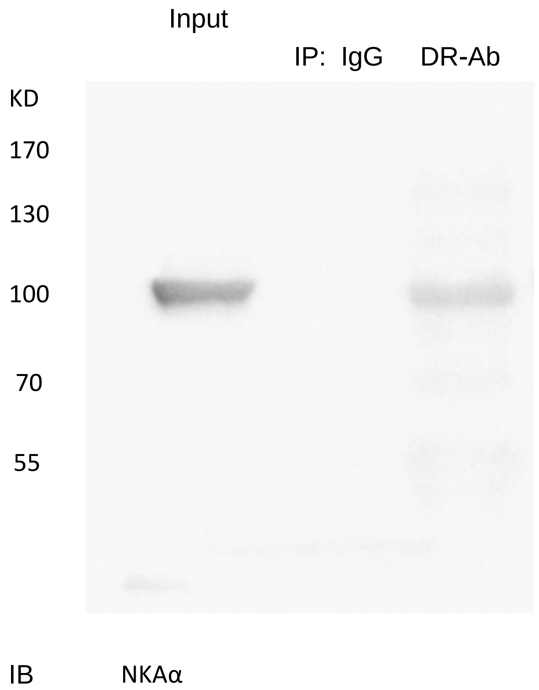

Fig 3b

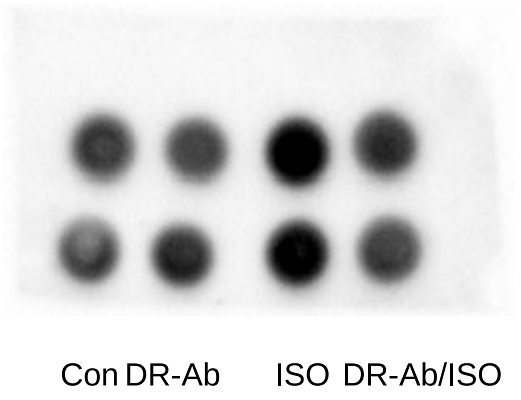

Fig 2e

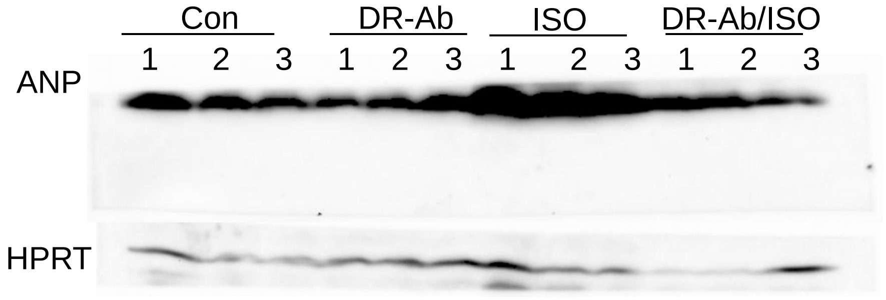

Fig 8f

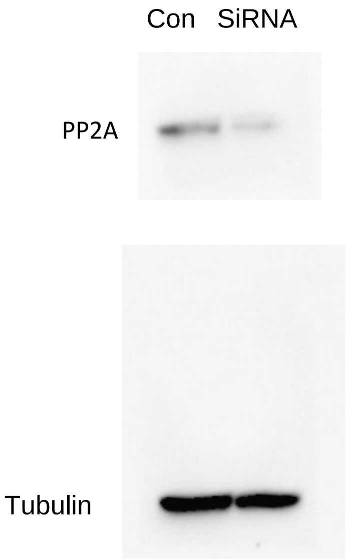

Fig 5b

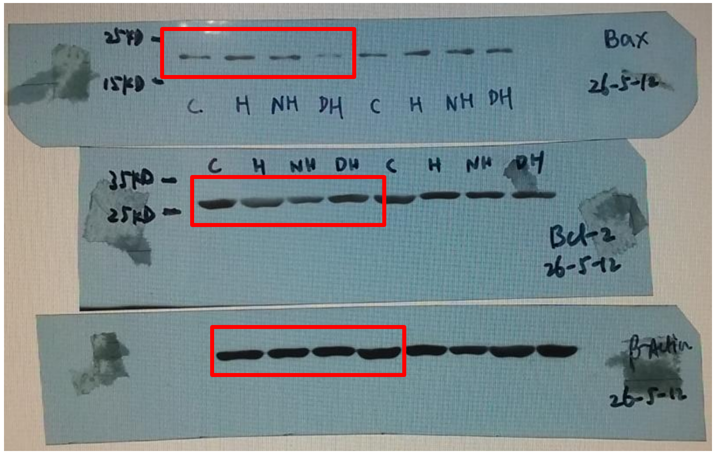

Fig 6b

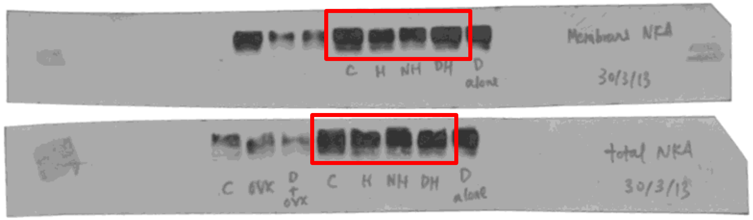

Fig 6c

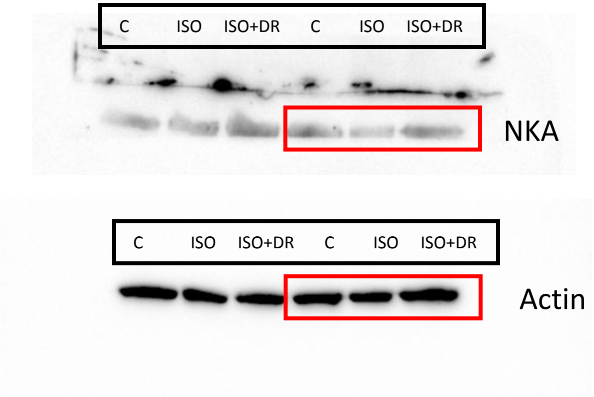

Fig 8a

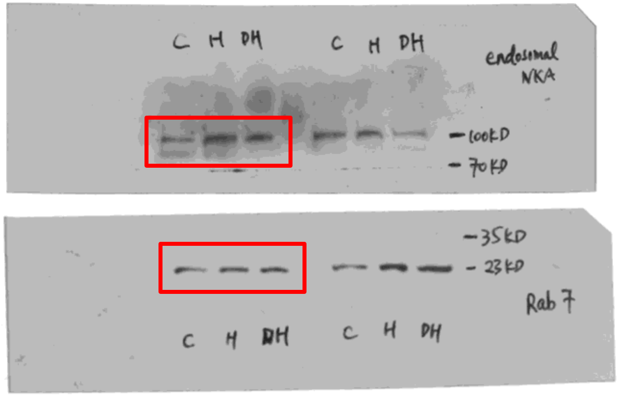

Fig 8b

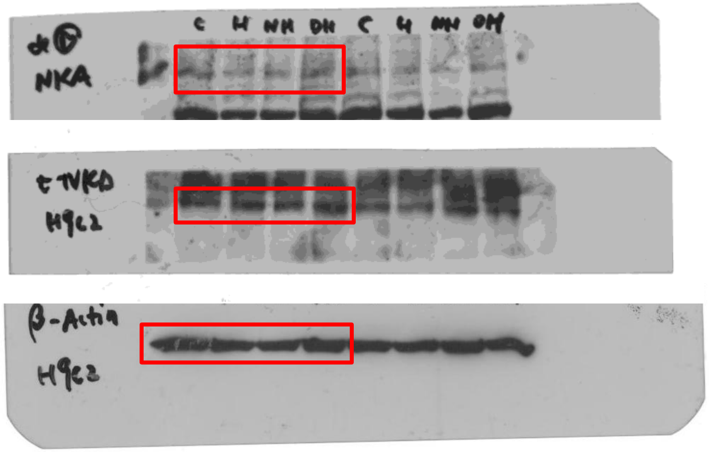

Fig 8c

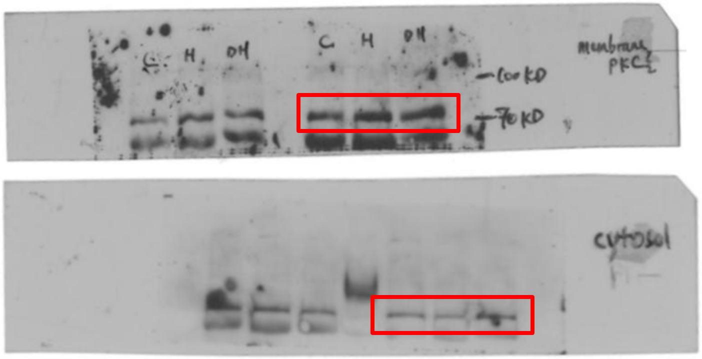

Supplement: Supplementary file 1 — Supplementary Information [file 41598_2018_31460_MOESM1_ESM.pdf]
